# Protocol for a Multicentric Cohort Study on Neonatal Screening and Early Interventions for Sickle Cell Disease Among High-Prevalence States of India

**DOI:** 10.3390/diagnostics15060730

**Published:** 2025-03-14

**Authors:** Suchitra Surve, Mahendra Thakor, Manisha Madkaikar, Harpreet Kaur, Shrey Desai, Rajasubramanium Shanmugam, Suman Sundar Mohanty, Apoorva Pandey, Anna Salomi Kerketta, Kapil Dave, Kalpita Ganpat Gawit, Lakshmana Bharathi Ramasamy, Oshin Warerkar, Prabhakar Kedar, Ragini Kulkarni, Saritha Nair, Nithin Rajamani, Anita Nadkarni

**Affiliations:** 1 Model Rural Research Health Unit (MRHRU), ICMR-National Institute for Research in Reproductive Health (NIRRCH), Dahanu 401601, Maharashtra, India; surves@nirrch.res.in (S.S.); kulkarnir@nirrch.res.in (R.K.); 2 ICMR-National Institute for Implementation Research on Non-Communicable Diseases (NIIR-NCD), Jodhpur 342005, Rajasthan, India; mahendra15519@gmail.com (M.T.); ssnimr@gmail.com (S.S.M.); 3 ICMR-National Institute of Immunohaematology (NIIH), Mumbai 400012, Maharashtra, India; madkaikarmanisha@gmail.com (M.M.); oshinwarerkar@gmail.com (O.W.); kedarps2002@yahoo.com (P.K.); nithinrajamani.icmr@gmail.com (N.R.); 4 ICMR-Indian Council of Medical Research Headquarters, New Delhi 110029, Delhi, India; kaurh.hq@icmr.gov.in (H.K.); apoorva.icmr@gmail.com (A.P.); nairs@icmr.gov.in (S.N.); 5 Society for Education, Welfare, and Action (SEWA)-Rural, Jhagadia 393110, Gujarat, India; sdesai1977@yahoo.com (S.D.); kapil.dave88@gmail.com (K.D.); 6 ICMR-National Institute of Research in Tribal Health (NIRTH), Jabalpur 482003, Madhya Pradesh, India; raja.rmrct@gmail.com; 7 ICMR-Regional Medical Research Centre (RMRC), Bhubaneswar 751023, Odisha, India; annasalomi@gmail.com; 8 ICMR-Centre for Research Medical & Health Centre (CRMCH), Chandrapur 442406, Maharashtra, India; drkalpita3@gmail.com; 9 Nilgiri Adivasi Welfare Association (NAWA), Kotagiri 643217, Tamil Nadu, India; nawaktg@gmail.com

**Keywords:** sickle cell disease, newborn screening, tribal population, haemoglobinopathies, early interventions, comprehensive care

## Abstract

**Background:** Sickle cell disease (SCD) is consequently associated with increased rates of infant and childhood morbidity and mortality. Therefore, early detection is a crucial aspect of managing SCD to mitigate complications and improve health outcomes for SCD children. Neonatal screening is the primary method for identifying newborns with SCD, enabling early diagnosis, family screening, and comprehensive medical care. The protocol presented in this paper describes a study aimed at screening newborns for SCD in high-prevalence SCD states of India to understand the magnitude of the problem and the benefits of early comprehensive care along with the genotypic and phenotypic correlation. **Methods:** A prospective cohort study will be conducted across seven sites in six states of India (Rajasthan, Odisha, Tamil Nadu, Maharashtra, Madhya Pradesh, and Gujarat), having a high prevalence of SCD. The cord blood or heel prick samples of all the live-born babies delivered within the facilities of selected regions will be collected for screening SCD by HPLC (High-Performance Liquid Chromatography). All the sickle cell homozygous (SS) babies will be confirmed at 6 weeks for Sickle genotype along with cascade screening. Further, SS babies will be followed up from six weeks up to five years of life with initiation of folic acid, antibiotic prophylaxis, and hydroxyurea treatment at appropriate times. **Results:** The protocol aims to lay the groundwork for the smooth implementation of newborn screening programs and effective follow-up strategies. **Conclusions:** It will pave the way for developing a strategic framework for implementing newborn screening programs for haemoglobinopathies in India.

## 1. Introduction

Sickle cell disease (SCD) is a worldwide health problem and has a very widespread geographical distribution including most of Africa, the Middle East, the Indian Subcontinent and parts of the Mediterranean [1]. The prevalence of the sickle cell trait in India exhibits significant variation among different tribal and social groups, with heterozygote frequencies ranging from 1–40% in many tribal populations [2]. However, community screening efforts in central India have revealed that the prevalence rates for SCD vary across various social categories, with 13.0% in the scheduled castes (SCs), up to 24% in the scheduled tribes (STs), 3.4% in the other backward caste (OBC) populations, and a higher prevalence of up to 33% among the populations of eastern Maharashtra [2,3,4,5]. The higher prevalence of SCD is consequently associated with increased rates of infant and childhood morbidity and mortality among Indian tribal communities, further aggravated by their limited access to healthcare services [6].

This genetic condition is characterized by the production of abnormal haemoglobin S and leads to a variety of severe health complications. When diagnosis and interventions are delayed, SCD can rapidly become a silent yet lethal condition. Children often succumb to sudden bacterial sepsis or acute splenic sequestration crisis (ASSC) within their initial years, and unfortunately, these fatal complications frequently occur before the infant’s condition of SCD is recognized by families or healthcare providers [7,8,9,10,11,12].

Therefore, a crucial aspect of managing SCD is early detection, which can significantly reduce morbidity and mortality in affected individuals. Further, studies have shown that early interventions, such as prophylactic penicillin and anti-pneumococcal vaccination, can dramatically reduce the risk of severe infections and death in young children with SCD [13]. Neonatal screening is a primary method for identifying newborns with SCD, enabling early diagnosis, family screening, and comprehensive medical care [11]. Hence, it is crucial to develop and implement a strong and efficient neonatal screening program to mitigate these complications and improve health outcomes for children suffering from SCD.

Neonatal screening is conducted either by collecting blood from the umbilical cord or by a heel prick. Three laboratory techniques, namely isoelectric focusing (IEF), haemoglobin electrophoresis, and high-performance liquid chromatography (HPLC), have mainly been used for neonatal SCD screening. IEF and/or HPLC are now being used in most newborn screening programs worldwide [14,15]. Molecular diagnosis by PCR is also recommended for mutation analysis for newborn screening [16,17].

Recently, several point-of-care (POC) testing devices have been developed based on solubility testing, differential erythrocyte density, wicking of HbS and HbA through filter paper, and polyclonal antibody-based capture immunoassay [18,19]. HemoType SC (Silver Lake Research Corporation, Irwindale, CA, USA) is one such POC test utilizing a qualitative lateral flow immunoassay method to detect the presence of HbA, HbS, and HbC with monoclonal antibodies [20,21], which has been demonstrated to be an affordable and rapid POC solution, particularly suitable for use in remote areas [22].

Currently, the national programme for prevention and management of sickle cell disease in India recommends screening for SCD among tribal populations as well as non-tribal people in selected geographies from high-prevalence and tribal states of India with aim of subsequent expansion to include all states across the country. For SCD newborn screening, the program advocates the use of HPLC. However, point-of-care (POC) tests validated by the Government of India are also recommended for mass screening and confirmatory diagnosis [23].

This paper describes a study protocol of a newborn sickle screening program across seven sites in six states of India, having a high prevalence of SCD. The protocol has two main components, viz., early screening and follow-up of the SCD babies. Through this, the study aims to understand the magnitude of the problem, and benefits of early comprehensive care and also evaluate the role of genetic modifiers in disease severity. 

The primary outcomes will help to assess the impact of early interventions on patient health and survival rates, as well as address the challenges and barriers to implementing such a program for scaling up.

## 2. Study Design

This will be a prospective cohort study focusing on neonatal screening for SCD and the effect of early interventions in the management of the SCD disease and has been designed in line with STROBE checklists [24]. It will also assess the challenges in implementing such a program, which will be determined by qualitative methods. 

## 3. Detailed Procedure

### 3.1. Study Setting

**Figure 1:**
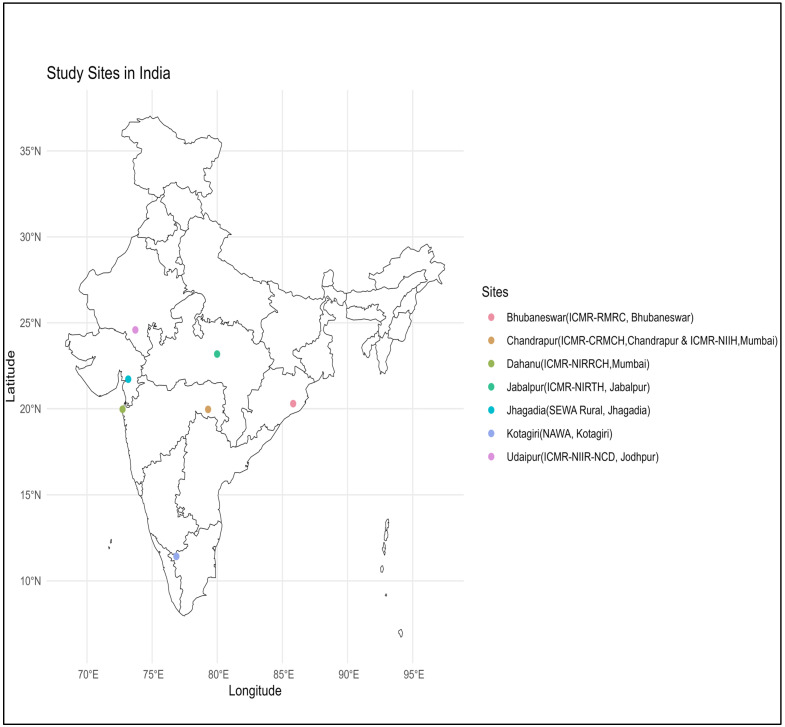
Study sites in India.

The study will be conducted across seven centres in India having high prevalence rates of SCD, as depicted in Figure 1, viz., Udaipur in Rajasthan, Bhubaneswar in Odisha, Kotagiri in Tamil Nadu, Dahanu, Chandrapur, and Gadchiroli in Maharashtra, Jabalpur in Madhya Pradesh, and Jhagadia in Gujarat. The selected areas encompass several tribal communities with limited access to healthcare facilities, which presents a unique challenge for neonatal screening and early intervention.

### 3.2. Study Population, Recruitment, and Sample Size

The study will be conducted across seven sites in six states of India, having a high prevalence of SCD (Figure 1). Each centre will identify a medical college/tertiary level hospital/district hospital/community health centre for the study. Considering the prevalence of 1% SCD and an average of 10% sickle cell trait, approximately 2000 newborns delivered at the facilities of selected centres will be required to be screened per year over five years to identify 100 SCD newborns during the study period, resulting in a cohort of approximately 700 SCD newborns at seven centres representing diverse genetic and socio-cultural settings.

### 3.3. Study Framework

The study will be implemented over five years, in three phases, viz., the preparatory phase, the implementation phase/data collection phase including screening and interventions, and the final phase of data analysis.

### 3.4. Preparatory Phase

During the initial preparatory phase of three months, administrative approvals will be obtained from the state and district health officials, followed by meetings with the gatekeepers of the community, such as the Sarpanch, local leaders, healthcare workers (ASHAs), and capacity-building of all stakeholders involved in the newborn screening at the centres, in addition to the development of questionnaires/Clinical Record Forms (CRFs). The capacity building will be conducted at three further levels, i.e., level I, encompassing orientation-cum-training workshops for all the investigators and staff (from each of the seven sites) regarding operational guidelines for diagnosis and management of SCD and various aspects of the study protocol, followed by hands-on training on various Standard Operating Procedures (SOPs) on data collection and entry and collection and transport of blood samples. 

Preparation of educational material in local languages for doctors, nurses, paramedical staff, and health workers in tribal areas will also be carried out. The site coordinators in the study, in level II, will conduct training activities for medical officers, and staff nurses of district hospitals/primary health centres (PHCs) to identify cases and record the information. Level III will include the conduction of training for health care workers, i.e., Anganwadi workers, ASHAs, and Auxiliary Nurse Midwives (ANMs) on recording information and strengthening the follow-up. The roles of the study team will be defined as shown in Table 1 to delegate responsibilities at each level.

The questionnaires/CRFs comprise sections for baseline and follow-up information. The baseline section includes demographic details, newborn screening results (focusing on haemoglobin levels and type of haemoglobinopathy), and a thorough family history of haemoglobinopathies (Supplementary Material S1A). The follow-up component of the questionnaire will be extensive, covering aspects such as medical history specifically related to SCD complications, detailed laboratory investigations including complete blood count (CBC) and biochemistry tests, and a comprehensive record of interventions and immunizations (Supplementary Material S1B).

### 3.5. Implementation Phase (Screening and Intervention Protocol)

#### Study Participants: Eligibility Criteria

All the live-born babies delivered within the facilities of selected regions will be included in this study whereas stillbirths and intrauterine foetal deaths (IUFDs) will be excluded. The mothers will be sensitized at the Antenatal Clinic (ANC) about the study during their routine ANC visits followed by recruitment under the study at labour-ward after obtaining written informed consent. 

Neonatal screening will be conducted by collecting the cord blood or heel prick sample. 

Screening for haemoglobinopathies will be performed on the Hb-Variant-II testing system (Bio-Rad Laboratories, Hercules, CA, USA) using the beta thal short program to identify the presence of the sickle cell trait or disease and other Hb variants [14]. The HemoType SC, a bed-side test to diagnose the haemoglobin phenotypes such as sickle cell homozygous (SS), sickle cell trait (AS), and normal babies (AA), will be performed on the first 300 samples at each centre, which will be stored in a refrigerator at 4 °C and later transported on ice packs at 8 °C to ICMR-NIIH, Mumbai, for confirmation of diagnosis. 

### 3.6. Methodology of Genetic Modifiers

The confirmation of the sickle genotype and genetic modifiers of the disease severity like alpha thalassemia and Xmn-1 polymorphism analysis on all the sickle cell homozygous samples from each centre will be carried out at ICMR-NIIH, Mumbai. For molecular analysis, DNA will be extracted using a Qiagen Flexi Gene DNA kit (Qiagen, Hilden, Germany) and subjected to DDE1 PCR and RFLP analysis using PCO2F (5′ACAGGTACGGCTGTCATCAC 3′) and PCO4R (5′ CAACTTCATCCACGTTCACC 3′) primers to determine the genotype (homozygous/heterozygous). The homozygous samples will be tested for α-thalassemia mutations using LIS-F (5′-ATACCATGGTTACCCCATTGAGC-3′), LIS-R (5′-AGGGCTCATTACATGTGGACCC-3′), α2/3.7-F (5′-CCCCTCGCCAAGTCCACCC-3′), α2-R (5′-AGACCAGGAAGGGCCGGTG-3′), 3.7/20.5-R (5′-AAAGCACTCTAGGGTCCAGCG-3′), 4.2-F (5′-GGTTTACCCATGTGGTGCCTC-3′), 4.2-R (5′-CCCGTTGGATCTTCTCATTTCCC-3′) primers. For this, a commercially available multiplex PCR kit (Qiagen, Hilden, Germany) will be used. Homozygous samples will also be investigated for XmnI polymorphism in the Gγ globin gene by amplification of a fragment containing the polymorphic site followed by digestion with the enzyme XmnI. OBG1 (5′AACTGTTGCTTTATAGGATTTT3′) and 1BG1 (5′AGGAGCTTATTGATAACCTCAGAC3′) will be used. Further, heterozygous samples will be screened for any α globin gene variants (non-deletion), for which DNA sequencing will be performed on an ABI 3730XL genetic analyser (Applied Biosystems, Foster City, CA, USA).

### 3.7. Family Screening, Parental Education, and Genetic Counselling

**Figure 2:**
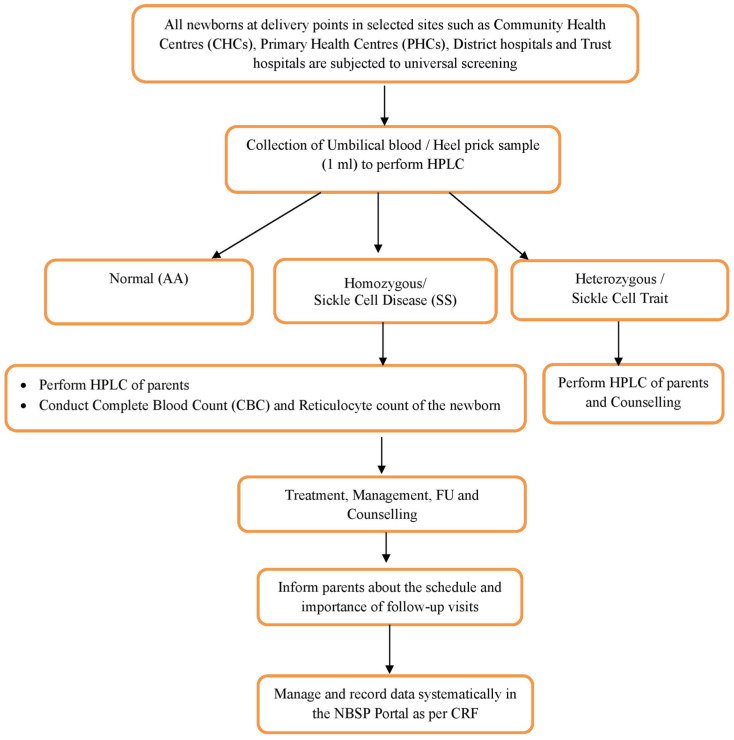
Study flow chart for protocol for a multicentred cohort study on neonatal screening and early intervention in SCD among high-prevalence states of India.

The parents along with their SS and AS newborns will be called for the first follow-up at 6 weeks corresponding to their immunization visit for confirmation of diagnosis along with cascade screening. The parents will be provided with information regarding the care of SCD children at home. They will be trained in spleen palpation, identifying early danger signs of complications, understanding the importance of hydration, regular follow-up, and medications as well as nutritional intake. The parents will further be counselled about the disease and risk for future pregnancies. Genetic counselling will be provided, emphasizing preventive strategies, including prenatal diagnosis, to avoid the birth of SCD babies in future. SS babies will be further followed up from six weeks to up to five years of life as per the schedules mentioned in the workflow of recruitment and follow-up (Figure 2). Follow-up beyond the study period will be integrated with the existing facilities of the selected centres for sustainability.

### 3.8. Treatment and Follow-Up Strategy

**Figure 3:**
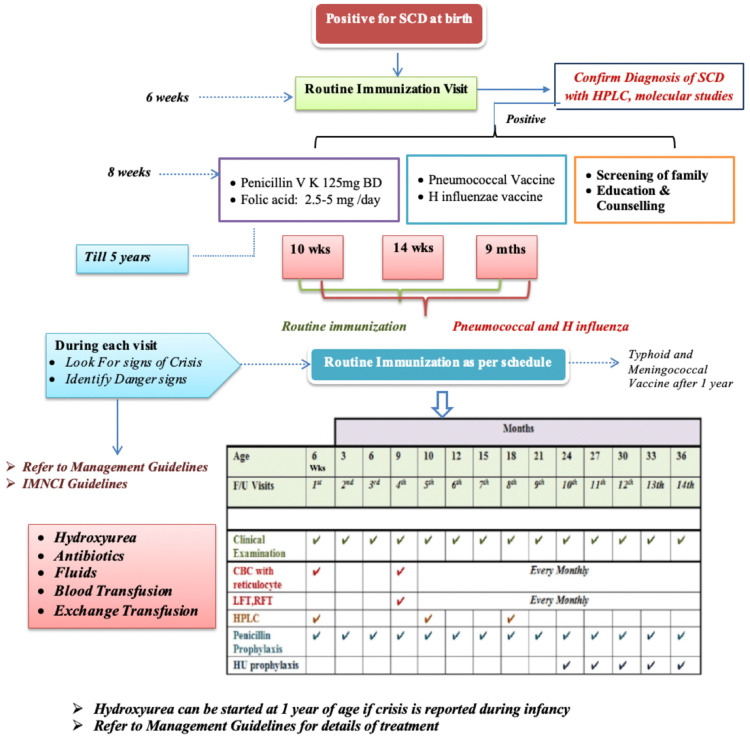
Treatment and follow-up protocol neonatal screening and early intervention in sickle cell disease among high-prevalence states of India.

During follow-up, detailed information about crisis events, illness history, and clinical examinations including weight, height, and head circumference will be recorded in the structured proforma (Supplementary Material S1). As per the management guidelines for SCD, formulated based on the existing National Health Mission (NHM) Guidelines, combined with clinical expertise (Supplementary Material S2 and Figure 3) [25], penicillin prophylaxis will be initiated from three months as 65 mg BD till one year; 125 mg BD for two years; then 250 mg BD after two years till the age of five years for SCD children. Amoxicillin (20 mg/kg/day) will be initiated for children who are allergic to penicillin as an alternative. Folic acid supplementation (2.5 mg for <one year of age and 5 mg daily for ≥one year of age) will also be initiated, as per standard of care treatment.

Conjugated pneumococcal (PCV) and *H. influenzae* B vaccination will be performed by merging with the existing vaccination schedule, as per NHM Guidelines [25]. Other additional immunizations will include unconjugated Pneumococcal Polysaccharide Vaccine (Pneumovax 23) for children >two years. 

Hydroxyurea (HU) will be initiated at 10–15 mg/kg/day [25] and will be escalated gradually by 5 mg/kg every 4–6 weeks only in definite indications (if there is no response or the child is still symptomatic, or if the child has a crisis and cannot be treated with a regular transfusion regimen) up to a maximum of 35 mg/kg/day. The baseline CBC with differential and reticulocyte counts, as well as liver and renal function tests, will be carried out before the initiation of HU. Serum B-12 and folate (to rule out HU-related macrocytosis), as well as serum iron, total iron binding capacity (TIBC), and ferritin (to rule out Fe-deficiency) tests, will be carried out if required based on the clinical evaluation.

Essential Monitoring during Hydroxyurea:

- CBC and reticulocyte count at least every 4 weeks, aimed at maintaining ANC > 2 × 10^9^/L and platelet count ≥80 × 10^3^/L;
- In cases of adverse events like neutropenia (<1000 cells/µL) or thrombocytopenia (<80 × 10^3^/L), HU will be stopped and monitoring of CBC will be performed weekly.

When the counts recover, the treatment will be restarted at 5 mg/kg/day lower than the previous dose on which the patient had cytopenia. Once the HU dose is stabilised, CBC with differential and reticulocyte counts every 3 months along with HPLC, alanine transaminase (ALT), aspartate aminotransferase (AST), blood urea nitrogen (BUN), and serum creatinine every 6 months will be performed. An increase in MCV and HbF levels will be used as evidence for compliance.

### 3.9. Strategies for Adherence to Follow-Up Treatment:

The parents will be asked to bring empty packets of the medicines. Also, the ASHAs, ANMs, and field staff will conduct extensive follow-ups to check treatment adherence. 

Clinical Data Collection, Management and Follow-Up will be conducted considering the following parameters: 

Baseline Assessment at Neonatal Screening:

- Demographics: Details of the newborn, viz., address, caste, age, sex, birth weight, antenatal details, and sickle cell status of parents;
- Screening Details: Date and time of the neonatal screening including repeat screenings and specific haemoglobinopathy screening results (e.g., HbA0, HbF, HbA2, HbS levels);
- Family Haemoglobinopathy History: Analysis of haemoglobinopathy status of family members (parents and siblings), based on HPLC reports.

Follow-Up Data Collection:

- Anthropometric Measurements: Regular measurements of weight, height, head circumference, and other relevant physical parameters;
- Medical History: Detailed recording of any complications related to SCD including hospitalization frequencies and reasons thereof;
- Laboratory Investigations: Routine and specific tests such as CBC, liver and kidney function tests, and HPLC evaluation;
- Treatment Records: Documentation of all medications prescribed for SCD management including any adverse reactions;
- Hydroxyurea: Initiation among SCD babies and assessing improvement in clinical manifestation post initiation of HU;
- Blood Transfusion History: History of hospitalizations specifically for blood transfusion;
- Hospitalization History: History of hospitalisation for crisis or any other issue;
- Immunization History: Comprehensive record of vaccinations, with particular attention to those indicated in SCD.

All data from the screenings and subsequent follow-up interventions will be meticulously recorded in the predesigned questionnaire after obtaining consent and subsequently managed through an online portal developed solely for this study by ICMR. 

### 3.10. Qualitative Data Collection (Interviews with Delivering Staff and Parents of SCD Children)

A qualitative study will be conducted to understand the perceived challenges, barriers, and facilitating factors to implement the newborn screening program. In-depth in-person interviews using interview guides will be conducted by trained interviewers among the purposively selected study participants who provide written informed consent. The participants would include Principal Investigators, Doctors, Medical Social Workers, Nurses, and Lab Technicians engaged in the implementation of the newborn screening and parents/guardians of SCD children from each site. Approximately 15 participants would be interviewed from each of the sites. The final sample size will be determined based on the principle of data saturation. Each interview is expected to last approximately 40–60 min. Consent will be obtained from the participants. Interviews will be audio recorded if consented. The recorded interviews will be transcribed verbatim, ensuring anonymity, and later translated into English for coding and analysis.

### 3.11. Final Phase of Data Analysis

Descriptive Statistics: This will involve summarizing patient demographics, disease incidence, and prevalence rates. Disease incidence and prevalence rates will be calculated and the measures of central tendency (mean, median) and dispersion (standard deviation, interquartile range) will be used for continuous variables, while frequency distributions and proportions/percentages will be used to summarize categorical variables.

Inferential Statistics: This will involve the evaluation of the effectiveness of early interventions such as prophylactic treatment and vaccinations. The study will also examine the correlations between specific genetic markers and the severity of the disease, potentially uncovering genetic influences on patient outcomes. Chi-square or Fisher’s exact tests will be used to compare categorical outcomes, while Student’s t-tests or Mann–Whitney U tests will be used to analyse continuous outcomes. Associations between continuous variables will be examined using Pearson or Spearman correlation coefficients, depending on the distribution of the data. Multivariable logistic and linear regression models will be used to explore these relationships further, adjusting for potential confounders such as socio-economic and environmental factors.

Survival Analysis: This will be conducted to assess the long-term outcomes and mortality rates among newborns diagnosed with SCD using Kaplan–Meier curves, log-rank tests, and Cox proportional hazards regression models. 

Multivariate Analysis: To understand the multifactorial effect of an SCD and its progression, multivariate analysis will be employed. This will involve exploring the impact of various factors such as genetic, socioeconomic, environmental, and treatment-related variables on the progression and management of the disease.

Qualitative Data Analysis: Thematic analysis of the collected data will be conducted following the six-step qualitative data analysis process suggested by Braun & Clarke (2006) [26]. Data will be coded using inductive and deductive codes. Coding will be performed in NVivo-14. Two social scientists will code the data simultaneously and independently to ensure consistency and avoid investigator bias. The initial codes and themes derived from the data will be discussed with the team members to incorporate their perspectives and suggestions in the data analysis. The findings from the data will be presented in the form of narratives, figures, tables, and interactive diagrams. 

### 3.12. Dissemination of Findings

Necessary steps will be taken to integrate the care of diagnosed SCD babies into the National Program through the dissemination of findings to the respective District, State, and Central tribal affairs departments.

### 3.13. Bias

This could occur if participants or caregivers underreport or overreport symptoms, treatment adherence, or complications related to SCD. Further, severity of SCD may vary in different regions. The interpretation of clinical signs and laboratory results by different healthcare professionals might vary, leading to observer bias. There may be confounding variables that are not controlled for, such as socio-economic status, access to healthcare, and other environmental factors that might influence the outcomes of neonatal screening and early interventions in SCD. A multivariate analysis and logistic regression will be applied to study the association of various factors and outcomes in these children to address confounding and bias issues.

The project framework with respect to specific objectives, outcomes, and period of activities is depicted in Table 2.

## 4. Expected Results

The primary outcome will assess the impact of early interventions on patient health in terms of reduction in crisis episodes, need for blood transfusion and survival rates as well as addressing the challenges and barriers in implementing such a program.

## 5. Discussion

There is an urgent need to diagnose babies with SCD at birth to ameliorate complications through early interventions. Despite published guidelines, lapses in its implementation largely affect disease outcomes [27]. This protocol not only comprises newborn screening but also suggests expanding the management of newborns to a multi-dimensional level, namely family screening. Further, the genetic diversity within and between tribal populations might lead to variable disease expressions, making it challenging to draw definitive conclusions about the role of specific genetic modifiers [28].

Genetic counselling (including prenatal diagnosis to avoid further birth of sickle homozygous babies), immunization, and timely follow-ups (for interventions including prophylactic antibiotics, folic acid, and hydroxyurea) will improve the quality of life of not only these children and reduce their morbidity and mortality but also their families. It thus measures the impact of screening on the physical and social aspects of the child, with a provision of offering education to family members [27] and throws light on preventive aspects through screening of families for sickle homozygous and heterozygous babies. The study includes a longitudinal follow-up component which will monitor the infants diagnosed with SCD for early developmental milestones, health complications, and their response to interventions. This approach is designed to provide comprehensive insights into the reduction in morbidity as a result of early screening and intervention strategies. However, ensuring long-term compliance with treatment in a community-based setting is challenging given loss to follow-up cases [29] and considering the limitation of the duration of the study, the relatively short duration of follow-up may not capture the full spectrum of long-term health outcomes and complications related to the disease, particularly in the context of late-onset complications (e.g., organ damage, renal failure, stroke, retinopathy, etc.).

The present study will identify the challenges and barriers to implementing such a program which will help to devise a mechanism for engaging the health system and community at different levels for improved newborn screening, to include timely follow-up thereby reducing morbidity and mortality. 

Thus, the protocol defines a strategic three-phased framework for running the program envisaging training of medical officers and community health workers for strengthening follow-ups at the community level, and follow-ups of SCD babies corresponding to vaccination time points during early years of life to deliver specific interventions. Further, our study will help to assess the three-dimensional impact of SCD management for confirmation of diagnosis and family screening, timely initiation of preventive measures, and finally genetic counselling for parents of the affected babies through effective recruitment strategies as well as community-based participatory research [30].

## 6. Conclusions

The protocol aims to lay the groundwork for the smooth implementation of newborn screening programs and effective follow-up strategies, which can play a pivotal role in attaining the objective of eradicating sickle cell anaemia by 2047 from India [23]. It will serve as a replicable model for screening and follow-ups of infants with SCD across other regions of the country. This will pave the way for developing a strategic framework for implementing newborn screening programs for haemoglobinopathies in India.

## Figures and Tables

**Table 1:** Multidisciplinary study team.

Member of the Team	Role in the Team
Site Investigator	Supervision of project activities; data management.
Biostatistician	Data analysis.
Project Scientist	- Recruitment of participants and follow-up; - Conduct clinical and systematic examinations of newborns in the field; - Involved in overall management and clinical assessment of newborns; - Provide counselling to parents about clinic visits; - Submit blood samples to the laboratory and follow-up forms to Counsellors; - Receive lists of newborns who missed their visits.
Counsellor/Medical Social Worker	- Prepare a list of newborns who missed their last visit using a web application or tracking system; - Enter follow-up data into the web application.
Laboratory Personnel	- Collect blood samples for CBC, RFT, and LFT; - Test the blood samples for CBC, RFT, LFT and carry out HPLC.

**Table 2:** Project framework: specific objectives, outcomes, and period of activities.

Project Phase	Duration	Specific Activities	Expected Outcomes
Screening and Initial Data Collection	4.5–5 Years	- Newborn screening for SCD; - Establishment of the patient cohort; - Initial data collection on disease incidence and genetic modifiers.	- Identification of newborns with SCD; - Initial database of patient clinical profiles including genetic data.
Follow-Up and Comprehensive Care	5 Years or till completion of the study	- Regular follow-up appointments; - Clinical and haematological assessments; - Interventions (penicillin prophylaxis, pneumococcal vaccines); - Continuous data collection on clinical progression and treatment efficacy.	- Detailed clinical trajectory of the disease; - Data on the effectiveness of early interventions in reducing morbidity and mortality; - Insights into genetic modifiers’ influence on the disease.
Data Analysis and Reporting	6 Months	- Data analysis; - Evaluation of screening effectiveness; - Impact assessment of early interventions; - Preparation and publication of final report.	- Comprehensive analysis of screening and intervention outcomes; - Publication of findings; - Recommendations for national screening programs and comprehensive care frameworks.
Addressing Implementation Challenges	Throughout Project	- Identifying and documenting potential barriers and challenges in implementing the program; - Developing strategies to overcome these barriers; - Adjusting project strategies based on ongoing assessment of challenges.	- Effective management of identified challenges and barriers; - Improved adaptability and responsiveness of the program to real-world issues; - Enhanced sustainability and impact of the program.

## Data Availability

Not applicable as this is a protocol manuscript.

## Supplementary Materials

The following supporting information can be downloaded at: https://www.mdpi.com/article/10.3390/diagnostics15060730/s1, Supplementary Material S1A: Neonatal Screening for Sickle Cell Disease—Case record Form Baseline visit; Supplementary Material S1B: Neonatal Screening for Sickle Cell Disease- Case record Form—Follow-up visit; Supplementary Material S2: Management guidelines for Sickle Cell Disease.

## Abbreviations

SCD	Sickle Cell Disease
SC	Scheduled Caste
ST	Scheduled Tribe
OBC	Other Backward Caste
ASSC	Acute Splenic Sequestration Crisis
IEF	Electric Focusing
HPLC	High Performance Liquid Chromatography
PCR	Polymerase Chain Reaction
POC	Point Of Care
STROBE	Strengthening the Reporting of Observational Studies in Epidemiology
SOP	Standard Operating Procedure
PHC	Primary Health Centre
ASHA	Accredited Social Health Activist
ANM	Auxiliary Nurse Midwife
CBC	Complete Blood Count
IUFD	Intra-Uterine Foetal Deaths
SS	Sickle Cell Homozygous
AS	Sickle Cell Heterozygous/Trait
AA	Normal Babies
BMI	Body Mass Index
NHM	National Health Mission
PCV	Pneumococcal Conjugated Vaccine
HU	Hydroxyurea
TIBC	Total Iron Binding Capacity
ALT	Alanine Transaminase
AST	Aspartate aminotransferase
BUN	Blood Urea Nitrogen
MCV	Mean Corpuscular Volume
