# Supplementary material for: Protocol for a Multicentric Cohort Study on Neonatal Screening and Early Interventions for Sickle Cell Disease Among High-Prevalence States of India
Source: Diagnostics (Basel). 2025 Mar 14;15(6):730. doi: 10.3390/diagnostics15060730 (PMC11941327; doi:10.3390/diagnostics15060730)
Supplement: Supplementary file 1 [file diagnostics-15-00730-s001.zip › diagnostics-3353530-supplementary.pdf]

Assessment Of Neonatal Screening Approaches For Sickle Cell Disease And The Effect Of Early Intervention In Management Of The Disease In Tribal Populations

Case Record Form-Baseline Visit

Part-A

Instructions: 1. Part-A is to be filled for all newborn babies who undergo screening for SCD, irrespective of result of screening. 2. 1. Screening and registration of newborn in NBS cohort should happen as early as possible after birth preferably within 2 days of birth and not later then 28 days of birth. 3. Part-A can be filled by team member other than medical doctor when screening is done at a health facility.

How to fill this form: Question numbers are written in first column in left side. Questions are written in left side of the second column followed by response blocks: Following type of answers are required: 1) Numbers- one character in one box as there are boxes available for each character of responses. NO BLANK VALUE PLEASE PUT ZERO BEFORE NUMBER IF, BOXES ARE MORE. 2) Date: Date format id given whenever answer is date 3) For categorical variables, responses to be written in each box, Data Collector need to draw circle around the answer.

N1.	Centre ID/ Site ID								
N1.1	Participant ID								
		D	D	M	M	Y	Y	Y	Y
N2	Date of Birth of newborn baby								
N3	Gender	Female				Male			
N4	Date of newborn screening								
N5	Address: ______________________________								
**House No.:**	**Village:**	**Taluka:**	**District:**<br>**State:**						
N6	Primary Phone number of family								
N6.1	Secondary Phone number of family or ASHA (Name)________________								
N7	Name of Mother ______________________________<br>(First name) (Middle name) (Surname)								

N8	Name of Father ______________________ (First name) (Middle name) (Surname)									
N9	Gestational age at birth (Term of the baby at Delivery)	<37 Weeks (Pre-term)		> 37 weeks; (Term)						
N10	Mode of delivery:	Instrumental (Forceps)		LSCS		Normal				
N11	Sickle status of Mother	Not available	Normal	Trait		Disease				
N12	Sickle status of Father	Not available	Normal	Trait		Disease				
N11	Birth weight (kg; one decimal e.g 2.8)									
N12	Name of the Caste/Tribe:									
N13	Consanguinity among the parents			Yes		No				
N14.1	HPLC report available for newborn?			Yes		No				
N14.2	If HPLC report available,			Value in concentration						
				#	#	. #				
		HbA0								
		HbF								
		HbA2								
		HbS								
		Other								
		Type of Hemoglobinopathy								
		Impression								
N15	Interpretation of Newborn HPLC	Normal	Trait	Disease						
N16	Follow up date									
		D	D	M	M	Y	Y	Y	Y	

POC device results of newborn (mandatory for first 300 samples)		Yes	No
1. AA	2. AS	3. SS	

Signature of Investigator (with date)

Signature of Field worker (with date)

Name of the Investigator

Clinical Notes/Miscellaneous: ______________________________

Attach follow up form with this

Assessment Of Neonatal Screening Approaches For Sickle Cell Disease And The Effect Of Early Intervention In Management Of The Disease In Tribal Populations

Case Record Form-Follow up visit

Instructions: 1. This registration form is for those neonates who are newly diagnosed with sickle cell disease and to record information at the time of follow up visits. 2. This form is to be filled by a doctor or in active participation with a medical doctor. 3. The completed form is to be stored with PI and data entry into a software should preferably happen on same day. After data entry, this follow up form should be attached with baseline form of respective patient to maintain longitudinal record of a case. 3. The family should be given a medical record along with a card and patient education brochure. 4. Family should be requested to inform PI in case of migration, change in phone number or any clinical event.

F1.1	Central ID/Site ID					
F1.2	Participant ID					

F2	Name of newborn ____ (If name is not available write B/O…)<br>(First name) (Middle name) (Surname)
F3	Sex : Male Female

 Participant Id:____________________________

Following Table is to be filled once at 6 weeks after birth or first visit after confirmatory HPLC is done.

C1	Date of repeat newborn screening									
			D	D	M	M	Y	Y	Y	Y
C2	If HPLC report of newborn,					Value of concentration				
						#		#	. #	
			HbA0							
			HbF							
			HbA2							
			HbS							
			Other							
			Type of Hemoglobinopathy							
			Impression							
C3	Interpretation of Newborn repeat HPLC		Normal			Trait		Disease		
C4	Blood Group of child									
C5	Interpretation of Mother's HPLC	NA	Normal			Trait		Disease		
C6	Interpretation of Father HPLC	NA	Normal			Trait		Disease		
C7	Interpretation of Sibling 1 HPLC	NA	Normal			Trait		Disease		
C8	Interpretation of Sibling 2 HPLC	NA	Normal			Trait		Disease		
C9	Interpretation of Sibling 3 HPLC	NA	Normal			Trait		Disease		
C10	Interpretation of Sibling 4 HPLC	NA	Normal			Trait		Disease		

 Participant Id:_____________________________

Following questions are to be recorded at every follow up visit including first follow up visit. If any of the data is not available then write “NA”

D1	Follow up number	1	2	3	4	5	6	7	8
D2	Follow up date DD/MM/YYYY								
D3	Is Child alive? (Y/N) (If yes go to question no D7)								
D4	Date of the death								
D5	Cause of death								
D6	Verbal autopsy form filled?								
D7	Weight : (kg; one decimal e.g 9.8)								
D8	Length/Height (Cms)								
D9	Head circumference: (Cms)								
D10	Mid arm circumference (Cms)								
D11	Presence of pedal edema (Yes/No)								
D12	Growth<br>< 3 SD (SAM)<br>2SD-3SD (MAM)<br>>2SD (Normal)								
**Complications of SCD since last visit (including current visit) (Write number in following boxes. Pl do not write “Yes/No”)**									

 Participant Id:______________________________

D13	Number of Acute severe painful Crisis since last visit								
D14	Number of Acute mild painful Crisis since last visit								
D15	Number of Acute Chest Syndrome events since last visit								
D16	Number of Sepsis/Severe Pneumonia events since last visit								
D17	Number of Splenic sequestration events since last visit								
D18	Number of Acute neurologic events since last visit								
D19	Pallor at the time of visit								
D20	Number of times patient had icterus since last visit?								
D21	Number of Aplastic crisis since last visit								
D22	Bone deformities/ Avascular necrosis (AVN) since last visit?								
D23	Number of febrile illness since last visit								
D24	Number of Acute respiratory infection (ARI) events since last visit								
D25	Number of dactylitis events since last visit								
D26	Number of Hospitalization since last visit								
D26.1	Reason for hospitalization								

 Participant Id:_____________________________

	1) Sequestration crisis<br>2) Infection/Fever<br>3) Vaso occlusive crisis<br>4) Severe anemia (3mg/dl)<br>5) Other causes related to SCD<br>6) Causes other than SCD								
D27	Number of times blood transfusion done since last visit								
D28	Mention the spleen size (cm) during visit								
D29	Mention the liver size (cm) during visit								
	**Values of investigations (CBC, Retic count, LFTs, RFTs and HPLC is to be done at least once a year. Write “ND” if not done. Clinician may decide to do additional testing in between)**								
D30	Hb								
D31	WBCs (in thousand)								
D32	RBCs (in thousand)								
D33	HCT								
D34	MCV								
D35	MCH								
D36	MCHC								
D37	Platelets (in lac)								
D38	Reticulocytes (%)								
D38.1	RDW								

 Participant Id:____________________________

D39	HbA								
D40	HbA2								
D41	HbS								
D42	HbF								
D43	Any other abnormal Haemoglobin (Yes/No) (Name:________________)								
D44	S. Bilirubin total								
D45	S.Bilirubin indirect								
D46	S.Bilirubin Direct								
D47	SGPT								
D48	SGOT								
D49	S.Creatinine								
D50	Blood culture (Y/N)								
D51	If yes, report								
D52	Urine routine/culture (Y/N)								
D53	If Yes, report								
**Treatment/Management (Write about medicines prescribed by project medical doctor or outside doctor during follow up visit)**									
D54	Pain killers prescribed? (Y/N)								
D55	Antibiotic/ penicillin prescribed? (Y/N)								
D56	Folic acid prescribed ?(Y/N)								

D57	Hydroxyurea prescribed? (Y/N)								
D58	If yes, Hydroxyurea dose (mg/kg) (NA)								
D59	Any adverse reaction to Hydroxyurea? (Y/N) (go to question no D60 if answer is No)								
D59.1	Please write adverse reaction name<br>1) Neutropenia<br>2) Thrombocytopenia<br>3) Abnormal LFT<br>4) Abnormal RFT<br>5) Other (please specify)								
D60	Justify Adverse reaction								
D61	Next Follow up date								
D62	Data collector's name								
D63	Data collector's Sign								

Please write about immunization received by the child as it happens.

**Immunization History**															
	**Vaccine**	**Project / Government**	**Is given during this follow up?**<br>**Yes**	**No**	**NA**	**Date: (If Yes)**	**D**	**D**	**D**	**M**	**M**	**Y**	**Y**	**Y**	
V1	BCG														
V2	IPV1														

V3	Penta 1													
V4	Rotavirus 1													
V5	IPV 2													
V6	Penta 2													
V7	Rotavirus 2													
V8	IPV 3													
V9	Penta 3													
V10	Rotavirus 3													
V11	MR 1st dose													
V12	DPT Booster-1													
V13	MR 2nd dose													
V14	DPT Booster -2													
*In case of Pentavalent vaccine, pl mention date of vaccine given against DPT, Hept.B and HiB. OPV should not be administered to Sickle cell patients.														

**Immunization History**											
	**Vaccine**	**Project /**	**Is given during this follow up? Yes No NA Date: (If Yes)**	**D**	**D**	**D**	**M**	**M**	**Y**	**Y**	**Y**

		**Government**												
V15	PCV 13 dose-1													
V16	PCV 13 dose-2													
V17	PCV 13 dose-3													
V18	PCV 13 dose-4													
V19	PCV -23													
V20	Meningococcal conjugate vaccination													
V21	Serogroup B meningococcal vaccination													
V22	Typhoid (Vi polysaccharide)													

Signature of Investigator (with date) **Signature of Field worker (with date)**

Name of the Investigator (with date) **Signature of the Scientist C (with date)**

 Participant Id:____________________________

Guidelines for management of Sickle Cell Disease (SCD)

Newborns and Children

(Consensus of expert group meeting)

Abbreviations:

CNS-Central Nervous System

CVS-Cardiovascular System

RS- Respiratory System

P/A-Per Abdomen

BP-Blood Pressure

TPR-Temperature, Pulse, Respiratory Rate

CBC -Complete Blood Count

HPLC-High Performance Liquid Chromatography

SCD-Sickle Cell Disease

MRI-Magnetic Resonance Imaging

MRA- Magnetic Resonance Angiography

MRV- Magnetic Resonance Venography

ALT- Alanine Transaminase

HU-Hydroxyurea

PCV-Pneumococcal Conjugate Vaccine

CKD-Chronic Kidney Disease

ANC- Absolute Neutrophil Count

Hb-Hemoglobin

IVIG-Intravenous Immunoglobulin

BMT-Bone Marrow Transplant

PRBC-Packed Red Blood Cells

HCT-Hematocrit

TBV-Total Blood Volume

HSM-Hepatosplenomegaly

ESRD-End Stage Renal Disease

Approach to a patient of SCD

**Presentation of patient**	
**Symptoms**	Persistent pallor, pain (musculoskeletal, joint pain, crisis), fever, lethargy, jaundice, difficulty in breathing
**Signs**	varying degrees of anaemia, icterus, toxic looks, leg ulcers
**Significant history**	family, affected siblings, cast/ religion, ethnicity, consanguity

⇩

**General examination**	**Systemic examination**
Vitals: TPR, BP, % saturation O2	CNS: consciousness, orientation
Hydration status	CVS: any adventitious sounds
Pallor, icterus, cyanosis (ends of toes / fingers)	RS: air entry, pneumonia
Joints: pain severity, mobilisation etc	P/A: Hepatosplenomegaly, ascites

⇩

**Investigations**	
Basic Investigations	CBC, peripheral smear, reticulocyte count
Screening test	Solubility test, Sickling test
Advanced test	HPLC analysis, DNA tests for mutations
• Less than 1 year age: solubility test and HPLC are not preferred<br>• i/c/o Transfusion given to patient: do tests after 3 months only	

⇩

**Routine care and management of SCD patients**	
Frequency of monitoring	Refer table number 1
Frequency of referral to specialities	Refer table number 2
Management of a new case of SCD	Refer table number 3
Management of specific situation/ emergencies	Refer table number 4
Transfusion support to SCD patients	Refer table number 5
Parental Education	Refer table number 6

Table 1: Frequency of monitoring:

Examination	Age	Frequency
Physical and systemic	0-24 months	Every 15 days to 3 months
	>24 months	Every 3 months
**Laboratory tests**		
CBC/ platelet/ retic count	3- 24 months	Every 3 months
	>24 months	Every 3 months (monthly if HU)
Liver function tests	12 months	Annually (Every 3 months if on HU)
Renal function tests	12 months	Annually (Every 3 months if on HU)
Urine(Microalbuminuria)	>6 years	Annually
**Screening tests**		
Transcranial Doppler	2-18 years	Annually<br>(if borderline repeat every 4 months,<br>if abnormal repeat every 2-4 weeks<br>MRI if abnormal)
Pulmonary function	>6 years	Annually based on clinical course
Echocardiogram	>12 years	Annually or depending on symptoms

Table 2: Frequency of referral clinic visits:

Clinic	Age	Frequency
Dental Examination	3-5 years	Annually
Ophthalmology	5 years	Annually
Adolescent consulting	14 years	As needed thereafter

Table 3: Management of a new case of SCD:

It includes approaches as follows:

Penicillin prophylaxis (refer table 3.1)
Folic acid supplementation (refer table 3.2)
Immunization (refer table 3.3)
Hydroxyurea (HU) therapy (refer table 3.4)

Table 3.1: Penicillin prophylaxis:

➢ < 5 years – 65mg BD till 1 year; 125 mg BD till 2 years; then 250mg BD after 2 years till age of 5<br>➢ In some setting it may be given for longer e.g. splenectomy

Table 3.2: Folic acid supplementation:

• <1 year of age : 2.5 mg daily
• ≥ 1 year of age : 5 mg daily

Table 3.3 Immunization: (In addition to routine vaccines)

Vaccine	Age/ Frequency
**Pneumoccocal Vaccine (conjugate)**<br><br>**Pneumococcal Vaccine (Polysaccharide)**	Routine immunization: at 6, 14 weeks & booster at 9 months (PCV 10 and 13 are available)<br>If not vaccinated and age 2-5 years: 2 doses 8 weeks apart<br>If not vaccinated and age 6-18 years: one dose<br><br>If immunized for age:<br>Polysaccharide vaccine after 1 month of conjugate vaccine and booster after 5 years.
**H-influenzae vaccine**	As routine immunization:<br>Pentavalent: at 6, 10, 14 weeks & booster at 12-15 months.<br>If not vaccinated and 1-5 years of age:<br>2 doses 8 weeks apart<br>If unvaccinated and >5 years: one dose
**Typhoid Vaccine**	Conjugate vaccine can be given as early as 9 months, 2nd dose may be given after 2 years (WHO recommends single dose)
**Influenza vaccine**	Everyone >6 months, 2 doses one month apart followed by yearly dose (after the 2nd dose)<br>Upto 3 years, dose is 0.25 ml s/c<br>>3 years, dose is 0.50 ml s/c<br>(Quadrivalent influenza vaccine is also available)

Table 3.4: Hydroxyurea (HU) therapy:

1. HU will be initiated as per clinical evaluation by treating clinician at local govt facility
2. Treat with HU all children > 9 months of age and all adults confirmed with SCD.
3. Do regular monitoring with CBC (monthly) and HbF (3 monthly) as cytopenia is major side effect.
4. Discontinue HU in pregnant or breast-feeding females.

   Note: In situations where follow-up or monitoring is not possible; fixed low dose HU (10 mg/kg) without escalation can be considered.

Dosing:

- 10 – 15mg/kg/ day of HU as single morning dose (increase if no response or symptoms persist).
- Dose escalation by 5 mg/kg every 4–6 weeks only if clinically indicated upto maximum dose of 35 mg/kg.
- In CKD patient: 5-10 mg/ kg/day.

1. **Essential Monitoring:**
   - CBC and Reticulocyte count every 4 weeks to maintain ANC >1.5 x10 ^{9}/L and platelet count ≥80,000/µL.
   - If neutropenia (ANC <1500 cells/mm^3.) or thrombocytopenia (< 80,000): hold HU and monitor CBC weekly.
   - When the counts recover, restart the treatment at 5mg/kg/day lower than the previous dose on which the patient had cytopenia.
   - **Always check for toxicities of HU (Short term):**

**Common (>10 %)**	**Occasional (1-10%)**	**Rare (<1%)**	**Very rare (<<1%)**
**Dose dependant :**<br>Neutropenia<br>Reticulocytopenia<br>Leukopenia	Thrombocytopenia	Diarrhoea	Allergic reaction
**Non-dose dependant :**<br>Nail / skin hyperpigmentation	Anemia<br>Thinning of hairs<br>Nausea<br>Anorexia	Gastritis<br>Pancytopenia<br>Rash<br>Vomiting	**Leg ulcer**<br>Increase in creatinine<br>Increase in ALT

Table 4: Care in emergencies and special situations:

**Manifestations**	
Painful event	Dactylitis, pain in the sternum/ribs, pain in the long bones and joints, Rx- hydration, analgesia PCM/ pethidine, $NaHCO_3$.
Infection	Bacteraemia/sepsis, meningitis, osteomyelitis, pneumonia, malaria
Anaemia	Splenic sequestration, transient aplastic crisis, transfusion reaction
Organ damage	Stroke, acute chest syndrome, splenic infarction, renal papillary necrosis, priapism, bone infarct, multi-organ failure

Complications	Presentation	Treatment	Prevention
**Sequestration Crisis** (pooling blood in the spleen or liver)	marked pallor, fall in Hb, HSM, hypovolaemic shock, reticulocytosis	Transfusion support: avoid over-transfusion splenectomy in recurrent acute splenic sequestration.	If > 2 episodes: Splenectomy Hypertransfusion in malaria endemic regions
**Hyperhemolytic crisis**	sudden hemolysis with worsening anemia despite ongoing reticulocyte production	Methylprednisolone 500 mg/day for two days) and intravenous immunoglobulin (IVIG; eg, 0.4 g/kg daily for five days	
**Stroke**	Headache, vomiting, seizures, various sensory or motor neurological deficits: hemiplegia/paresis, paraplegia/paresis, monoplegia /hemiparesis, loss of hearing, etc.	Stabilization, support, monitoring of vital signs as necessary; maintain normal temperature, oxygenation, careful hydration, hydroxyurea MRI with MRV/MRA Transfusion: keep HbS<30%, Iron Chelation	Trans-cranial USG (2-16 years of age) Consider HU or Tx to keep HbS<30%)

**Priapism** prolonged painful penile erection, not associated with sexual stimulation	Stuttering lasts< 4 hr; resolves spontaneously<br>Major or fulminant, > 4 hrs<br>Onset: Mostly in the early hours of the morning and at nights	Stuttering:<br>Etilefrine 25mg/ day HS<br>to a maximum dose of 100mg<br>+/- Cyproterone	Referral to Urosurgeon for chronic priapism<br>Irrigation with drugs locally, shunt surgery, Exchange Tx
**Chronic Leg Ulcers**	Ulcers on medial or lateral malleolus of one or both ankles	Daily dressing, rest and elevation, treat infection, skin graft	Avoid walking and running, infection, scabs, osteomyelitis
**Infections**	Staph, strepto, Chlamydia, capsulated micro-organisms, salmonella, or other enteric pathogens etc. , parvovirus, H1N1 influenza, Zika virus, malaria and dengue	Blood and urine culture , CBC<br>empiric parenteral antibiotics<br>Penicillin VK 125 mg BD PO < 3 years<br>250 mg BD > 3 years. Erythromycin if allergic to penicillin.<br><br>T/t as per infective agent	Immunization with vaccines
**Hypersplenism**	Splenomegaly with anaemia, leucopenia, thrombocytopenia, with compensatory bone marrow hyperplasia for a sustained period and improvement after splenectomy	Splenectomy complete or partial	

**Acute Chest Syndrome**	Fever >38.5°C<br>Respiratory symptoms<br>chest X-ray (e.g. consolidation)	Admission, O2, exchange BT, Antibiotics, analgesics, bronchodilators, anticoagulation, hydration	Hydroxyurea therapy or hypertransfusion

Initial Treatment of Patient in Sickle Cell Crisis:

5. **Pain relief:**

Management of pain in SCD:

Degree of Pain	Opioid Naive	Opioid Tolerant
**Mild**	Adult:<br>Dihydrocodeiene tablet 30 mg 4 hrly plus Paracetamol 1g 4 hrly	Immediate-release oral formulation of stronger opiate e.g. morphine 4 hrly
	Children:<br>• Paracetamol 20mg/kg 4 to 6hrly with/without Ibuprufen 10mg/kg 8hrly Or diclofenac 1mg/kg 8hrly<br>• Dihydrocodiene 1mg/kg 8hrly	
**Moderate/Severe**	Adult:<br>• Diamorphine 2.5-5mg/4hrly s.c. Or morphine 5-10mg stat or pentazocine 15 mg 8-hrly.<br>• Inj diclofenac 75mg 1-2 hrly after opioids<br>• Inj tramadol 100mg 1-2 hrly after NSAIDs	• Injection of opiate diamorphine 10-20mg/2-4 hrlys.c.<br>• Injection of opiate e.g. pentazocine 30 - 60 mg 8-hrly
	Children:<br>• Oral morphine 0.4mg/kg or diamorphine 0.1mg/kg in IV infusion or IM/SC stat to immediate pain relief. Then maintenance: Slow release morphine 1mg/kg (rounded up to 5mg) every 12 hrs with oral morphine 0.3mg/kg every 3hrs as necessary. Or oral	

	morphine 0.4mg/kg Or pentazocine at appropriate doses<br>• Inj diclofenac 1mg/kg after opoids<br>• Inj tramadol 100mg 1-2 hrs after NSAIDs	

- Assess pain relief every 15-30 minutes
- Anti-emetics: procholorperazine 250 μg/kg t.d.s. or cyclizine 12.5-25 mg t.d.s.
- Monitor: pain, vital signs X 30 min until patient is stable; then every 2 hours
  Monitor opioid induced respiratory depression

6. **Optimal hydration:**

**Adults**	**Children**
1.5 L/m^2/day OR<br>3L/day	100 – 120 ml/kg/day<br>(can be given orally if there is no vomiting or if patient can drink that volume)

5% dextrose or Dextrose Normal Saline (DNS) is preferred. Avoid hypertonic fluid e.g.10% dextrose

7. **Urgent Referrals Criteria in case of the following:**

- Acute neurological symptoms
- Organ damage with SCD; sequestration/ aplastic/ hemolytic crisis
- Toxicity to hydroxyurea
- For exchange transfusion

8. **Chronic clinical complications of sickle cell disease and management:**

**Organ**	**Manifestations**	**Treatment**
Kidneys	Hyposthenuria, glomerular hyperfiltration, glomerulosclerosis, albuminuria, ESRD	ACE Inhibitors, hydroxyurea, dialysis, renal transplant
Heart/Lungs	Restrictive lung disease, elevated tricuspid jet velocity, pulmonary hypertension, restrictive cardiomyopathy	Bronchodilators, hydroxycarbamide, transfusion

Brain	Ischemic stroke, haemorrhagic stroke, silent infarction, neurological decline	Transfusion, hydroxycarbamide
Liver	Jaundice, pigmented gall stones	Cholecystectomy
Spleen	Infarction, hypersplenism	Splenectomy
Bones/skin	Avascular necrosis, leg ulcers	Physical therapy, cord decompression, wound care. Surgery
Eyes	Retinopathy	Laser therapy
Penis	Impotence, infertility	Surgery (if needed)

Indications for admission:

Acute illness requiring immediate medical care, including emergencies that need to be defined :-

a. Temperature >38 ^{0}C
b. Pain inadequately relieved by home measures (Crocin , Plenty of fluids)
c. Significant respiratory symptoms (cough, shortness of breath, chest pain)
d. Abdominal pain, distension, acute enlargement of spleen
e. Any neurological signs or symptoms, if any
f. Significant increase in pallor, fatigue, lethargy
g. Significant vomiting and diarrhoea

Table 5: Transfusion support:

- Avoid blood transfusion unless anemia is symptomatic
- Transfusion in SCD can be prophylactic to prevent or manage complications like stroke or only in the case of an acute life threatening complication
- Do at every Transfusion event: Blood group, cross match, consider extended blood typing (especially Rhesus, Kell, Duffy) or molecular genotyping
- Other recommendations of transfusion in SCD patients-
- Pre-storage leucodepletion or leucodepletion filters (3- 4log)
- Irradiation, if planning for BMT
- Immunize against hepatitis B
- Monitor for iron overload and start chelation therapy appropriately

1. **Simple transfusion:**

**Indications**	
1	Severe anemia (ie, Hb <5 g/dL),
2	Pre-operative transfusion to reduce complications of surgery
3	Hb <10 g/dl with symptoms of anemia
**Contraindications**	
1.	Patients with SCD complications whose Hb is 7 to 9 g/dL
2	Hb >10 g/dL and a HbS percentage >50 of total Hb

2. **Exchange blood transfusion (**automated apheresis or manual):

**Indications**
Multi-organ failure, Suspected stroke, Respiratory compromise, Acute chest syndrome
Regular: for prevention of stroke, acute chest syndrome, recurrent painful episodes

A full exchange transfusion rapidly lowers the HbS level to 30% or less and improves the hemoglobin level. In general, automated apheresis is preferred over manual.

Blood transfusion volume:

- Dose of red cells should be calculated to result in a Hb 10 g/dL or HCT 30%
- Children: Transfusion of 10 mL/kg will increase Hb 2.5 to 3.0 g/dL
- Adults : one unit of PRBC will increase the Hb by approximately 1 g/dL

Formula used for estimation of transfusion volumes:

1. **simple transfusion (mL) =** ([dHCT - iHCT] x TBV) ÷ RpHCT (where dHCT= desired % hematocrit, iHCT = initial % hematocrit, TBV = total blood volume in ml, RpHCT =% hematocrit of the replacement packed RBC)
2. **Manual partial exchange volume (mL)** = ([dHCT - iHCT] x TBV) ÷ (RpHCT - [(iHCT + dHCT) ÷ 2]

Table 6: Parental education:

a)	Genetic Counselling
b)	Sensitize to requirement of routine follow-up and monitoring
c)	Attention to fever and grade of fever
d)	Vaccination
e)	Recognition of pain
f)	Home remedy for pain (Crocin, Plenty of liquids)
g)	Need for hydroxyurea
h)	When to seek medical help
**i)**	Spleen palpation

References:

1. NHM Guidelines for Hemoglobinopathies in India 2016.
2. Roshan B. Colah, Malay B. Mukherjee, Snehal Martin, and Kanjaksha Ghosh Sickle cell disease in tribal populations in India. Indian J Med Res. 2015 May; 141(5): 509–515.

Abbreviations:

1. Severe anemia-Hb< 5 gms%
2. Neutropenia - absolute neutrophil count (ANC) of less than 1,500 cells/mm^3
3. Thrombocytopenis -Platelet Count less that 80,000 cells/mm^3
